# Health-related quality of life is related to COPD disease severity

**DOI:** 10.1186/1477-7525-3-56

**Published:** 2005-09-09

**Authors:** Elisabeth Ståhl, Anne Lindberg, Sven-Arne Jansson, Eva Rönmark, Klas Svensson, Fredrik Andersson, Claes-Göran Löfdahl, Bo Lundbäck

**Affiliations:** 1 Department of Respiratory Medicine and Allergology, University Hospital, SE-221 85 Lund, Sweden; 2 AstraZeneca R&D Lund, SE-221 87 Lund, Sweden; 3 The OLIN Studies, Department of Medicine, Sunderby Central Hospital of Norrbotten, SE-971 80 Luleå, Sweden; 4 Department of Respiratory Medicine and Allergy, University Hospital, SE-901 85 Umeå, Sweden; 5 Lung and Allergy Research, National Institute of Environmental Medicine, the Karolinska Institute, SE-171 77 Stockholm, Sweden

**Keywords:** Health-related quality of life, COPD, disease severity, epidemiological, Global Initiative for Chronic Obstructive Lung Disease (GOLD), St George's Respiratory Questionnaire (SGRQ)

## Abstract

**Background:**

The aim of this study was to evaluate the association between health-related quality of life (HRQL) and disease severity using lung function measures.

**Methods:**

A survey was performed in subjects with COPD in Sweden. 168 subjects (70 women, mean age 64.3 years) completed the generic HRQL questionnaire, the Short Form 36 (SF-36), the disease-specific HRQL questionnaire; the St George's Respiratory Questionnaire (SGRQ), and the utility measure, the EQ-5D. The subjects were divided into four severity groups according to FEV_1 _per cent of predicted normal using two clinical guidelines: GOLD and BTS. Age, gender, smoking status and socio-economic group were regarded as confounders.

**Results:**

The COPD severity grades affected the SGRQ Total scores, varying from 25 to 53 (GOLD p = 0.0005) and from 25 to 45 (BTS p = 0.0023). The scores for SF-36 Physical were significantly associated with COPD severity (GOLD p = 0.0059, BTS p = 0.032). No significant association were noticed for the SF-36, Mental Component Summary scores and COPD severity. Scores for EQ-5D VAS varied from 73 to 37 (GOLD I-IV p = 0.0001) and from 73 to 50 (BTS 0-III p = 0.0007). The SGRQ Total score was significant between age groups (p = 0.0047). No significant differences in HRQL with regard to gender, smoking status or socio-economic group were noticed.

**Conclusion:**

The results show that HRQL in COPD deteriorates with disease severity and with age. These data show a relationship between HRQL and disease severity obtained by lung function.

## Background

Chronic obstructive pulmonary disease (COPD) is a major cause of morbidity and mortality worldwide and is currently the fourth leading cause of death in the US [1]. It is a slowly progressive disease, characterized by lung function impairment with airway obstruction [2,3]. Common symptoms are cough, sputum production and shortness of breath. Smoking and different air pollutants, such as are well-known risk factors for COPD [3,2].

The prevalence of COPD varies considerably between countries and areas, from 3% in India [4] to 23% in the inner-city population of Manchester, UK [5]. The US National Health and Nutrition Examination Survey (NHANES) III survey puts the prevalence of COPD in the US at 7% [6]. The figure in Spain is similar, 9% [7]. In Sweden, the prevalence of COPD in those aged above 45 years was estimated to be 8% according to the British Thoracic Society (BTS) criteria and 14% according to the Global Initiative for Chronic Obstructive Lung Disease (GOLD) guidelines [8]. However, there are a considerable number of subjects with COPD who have not been diagnosed as such. In Europe and also in Sweden only one-quarter to one-third of those with COPD have been diagnosed as having COPD or with different labelling of the disease [8-11].

Over the past decade, more and more research on the development and validation of questionnaires has been undertaken to quantify the impact of disease on daily life and well-being from the COPD subject's point of view [12]. Health-related quality of life (HRQL), and preference-based HRQL instruments (utility instruments) are increasingly used in clinical studies. Although their use is established in many fields, such as oncology and gastrointestinal disease, questionnaires are rarely used as primary endpoints in randomised clinical studies of respiratory disease. One possible reason may be the lack of information about the patients' deterioration in HRQL when the disease progresses. The Medical Outcomes Study Short Form 36 (SF-36) and St George's Respiratory Questionnaire (SGRQ) are generic and disease-specific HRQL questionnaires, respectively [13,14]. The SF-36 has been used in a number of therapeutic areas, including COPD, while the SGRQ has been widely used in both COPD and asthma research. The EQ-5D is a generic, preference-based utility measure and has been used in a number of therapeutic areas [15].

The aim of the present study was to evaluate the association between HRQL and COPD stages using forced expiratory volume in one second as a percentage of predicted normal values (FEV_1 _% predicted) by means of two clinical guidelines for COPD, taking into account the influence on HRQL of age, gender, smoking status and socio-economic background. The association between HRQL and forced vital capacity as a percentage of predicted normal values (FVC % predicted) was also evaluated.

## Methods

### Study sample

A total of 202 subjects with COPD, recruited from a representative sample of the general population in northern Sweden, were invited; 176 subjects took part in this survey and data from 168 subjects were available [16]. The study cohort was derived from the Obstructive Lung Disease in Northern Sweden (OLIN) Studies [8,9], which has previously been described in detail [16].

### Procedure

After initial instruction from the administrator, a qualified nurse, the questionnaires were completed unaided by subjects in the order SF-36, SGRQ and EQ-5D. A few subjects did not complete all questionnaires.

#### Definition and severity of COPD

The subjects were divided into four severity groups according to FEV_1_% predicted (pre-bronchodilator) using two different guidelines: the updated version (not yet published) of the GOLD guidelines [3] and the BTS guidelines [2]. The definition and severity criteria are described in Table 1. Calculation of FEV_1_predicted normal values for FEV_1 _was based on the reference values from ERS guidelines. In addition, levels of FVC % predicted were also used in the analysis instead of COPD severity stages.

**Table 1:** Severity criteria of COPD

**Global Initiative for Chronic Obstructive Lung Disease, GOLD [3]: FEV_1_/FVC < 70%**		
I:	Mild COPD	FEV_1 _≥ 80% predicted
II:	Moderate COPD	FEV_1 _50- < 80% predicted
III:	Severe COPD	FEV_1 _30- < 50% predicted
IV:	Very severe COPD	FEV_1 _< 30% predicted
**British Thoracic Society, BTS [2]: FEV_1_/VC < 70% and FEV_1 _< 80% predicted**		

I:	Mild COPD	FEV_1 _60- < 80% predicted
II:	Moderate COPD	FEV_1 _40-59% predicted
III:	Severe COPD	FEV_1 _< 40% predicted

A group labelled BTS stage 0 was created for subjects with FEV_1 _≥ 80% predicted: i.e. identical with mild COPD according to the GOLD criteria.

### HRQL questionnaires

#### Short Form 36

The most widely used generic questionnaire, the Medical Outcomes Study Short Form 36 (SF-36), has been widely accepted in recent years as the best generic HRQL measurement. It contains 36 items divided into eight domains: Physical Functioning (PF), Role-Physical (RP), Bodily Pain (BP), General Health (GH), Vitality (VT), Social Functioning (SF), Role-Emotional (RE) and Mental Health (MH). These domains create a profile of the subject. Two summary scores can also be aggregated, the Physical Component Summary (PCS) and the Mental Component Summary (MCS). Scores range from 0 to 100, with higher scores representing better HRQL.

#### St George's Respiratory Questionnaire

The best-known and most frequently used disease-specific HRQL questionnaire for respiratory diseases, is the St George's Respiratory Questionnaire (SGRQ) [14,17]. The SGRQ is a standardized, self-administered questionnaire for measuring impaired health and perceived HRQL in airways disease. It contains 50 items, divided into three domains: Symptoms, Activity and Impacts. A score is calculated for each domain and a total score, including all items, is also calculated. Each item has an empirically derived weight. Low scores indicate a better HRQL. Recent publications by the developer (PW Jones) have confirmed that the minimal important difference relevant to the patients (MID) is 4 on a scale of 0 to 100 [18,19].

#### EQ-5D

The EQ-5D is a generic, preference-based utility questionnaire and consists of two parts, the EQ-5D VAS and the EQ-5D index. The EQ-5D has been used in a number of therapeutic areas and contains a vertical rating scale from 0 to 100 (EQ-5D VAS), with 0 = death/worst possible health and 100 = best possible health. The EQ-5D index is a five-item questionnaire ranging from 0 to 1. The items consist of mobility, self-care, usual activity, pain/discomfort and anxiety/depression. Each item has three levels: no problem, some problem and severe problem [15]. For the EQ-5D index, 0.03 has been regarded as the MID [20].

### Statistical analysis

Statistical analysis was performed using an analysis of covariance model with HRQL scores as dependent variable. Three different approaches to analysis were performed using different classification of severity of COPD from GOLD and BTS guidelines. This classification was used as factor in the analysis. In all cases age, gender, smoking status and socio-economic background was used as covariates. These variables showed sign of influence on the HRQL measures and for the sake of comparability a unified model was selected for the analysis. An additional classification of severity based on FVC % predicted normal was also investigated with the same model with classification into four groups: stage I: > 95%, stage II: 95-81%, stage 3: 80-66% and stage IV; < 66%. These levels were chosen to have approximately equal number of patients in each group. Data presented in tables are adjusted least-square means from the adopted model.

## Results

### Subject characteristics

The mean age of the 168 subjects (70 women) was 64.3 years (range: 28–80 years). In the six age groups (the lowest < 45 and the highest > 79 years), 57 of the subjects were smokers and 85 were ex-smokers. Three socio-economic groups were identified (manual employees, non-manual employees and unemployed including housewives). Of the 138 'employees', 65 were still working and 73 had retired, and of these, 40 had retired before the normal age of retirement. Table 2 shows the subjects' characteristics.

**Table 2:** Subject characteristics

**Characteristic**	**Mean data (range)**
n, total number agreed	176
Men/women n = 168	98/70
Mean age, years (range) n = 168	64.3 (28–80)
FEV_1_, L (range) n = 159	1.76 (0.46–4.12)
FEV_1 _% predicted (range) n = 159	62 (18–118)
Smoking status n = 171	Smoker, n = 57 Non-smoker, n = 29 Ex-smoker, n = 85
Socioeconomic group n = 174	Manual employees, n = 78 Non-manual employees, n = 60 Unemployed incl. housewives, n = 36

### HRQL in relation to COPD severity according to GOLD

The differences in SF-36 PCS between the four severity groups were statistically significant (p = 0.0059). The scores for SF-36 (PCS) were 42 in the stage I group and 29 in the stage IV group. The corresponding scores for SF-36 MCS were 55 and 48 in stages I and IV respectively (p = 0.19) (Table 3).

**Table 3:** Health-related quality of life scores, adjusted mean values (± SD) – GOLD criteria

**Scale**	**FEV_1 _% predicted**				
	**≥ 80% Stage I n = 26**	**79-50% Stage II n = 91**	**49-30% Stage III n = 33**	** < 30% Stage IV n = 9**	**p-value (all stages)**
SF-36 PCS	42(12)	42(12)	40(10)	29(12)	0.0059
SF-36 MCS	55(8)	51(11)	52(12)	48(18)	0.19
SGRQ Total	25(20)	32(20)	36(20)	53(23)	0.0005
EQ-5D VAS	73(21)	65(24)	62(21)	37(28)	0.0001
EQ-5D index	0.84(0.15)	0.73(0.23)	0.74(0.25)	0.52(0.26)	0.0008

**Figure 1:**
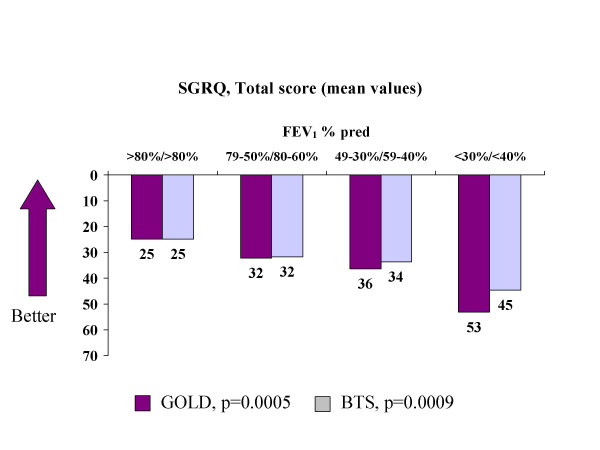
**SGRQ, Total score (adjusted mean values) in GOLD and BTS stages. **p-values by test for trend.

There was also a statistically significant difference in the SGRQ scores between the severity groups (p = 0.0005). The severity grades affected the level of SGRQ Total as follows: stage I: 25, stage II: 32, stage III: 36 and stage IV: 53 (Table 3, Figure 1).

The scores for EQ-5D VAS were 73 in stage I and 37 in stage IV (p = 0.0001). EQ-5D index showed the following scores: stage I: 0.84 and stage IV: 0.52 (p = 0.0008) (Table 3).

### HRQL in relation to COPD severity according to BTS

The scores for SF-36 (PCS) were 42 in the group labelled stage 0 and 35 in stage III (p = 0.032). The corresponding scores for SF-36 MCS were 55 and 50 in stages 0 and III, respectively (p = 0.29) (Table 4).

**Table 4:** Health-related quality of life scores, adjusted mean values (± SD) – BTS criteria

**Scale**	**FEV_1 _% predicted**				
	**≥ 80% Stage 0 n = 26**	**79-60% Stage I n = 63**	**59-40% Stage II n = 47**	** < 40%Stage III n = 23**	**p-value (all stages)**
SF-36 PCS	42(12)	43(11)	40(13)	35(11)	0.032
SF-36 MCS	55(8)	50(10)	54(11)	50(15)	0.29
SGRQ Total	25(20)	32(20)	34(19)	45(22)	0.0023
EQ-5D VAS	73(21)	68(20)	60(28)	50(25)	0.0007
EQ-5D index	0.84(0.15)	0.74(0.21)	0.72(0.28)	0.63(0.25)	0.0041

The severity grades affected the level of SGRQ Total scores as follows: stage 0: 25, stage I: 32, stage II: 34, and stage III: 45. There was a statistically significant difference in the SGRQ Total scores between the severity groups (p = 0.0023) (Table 4, Figure 1).

The scores for EQ-5D VAS were 73 in stage 0 and 50 in stage III (p = 0.0007). The EQ-5D index scores were 0.84 and 0.63 in stages 0 and III, respectively (p = 0.0041) (Table 4).

### Influence of age, gender, smoking status and socio-economic group

**Figure 2:**
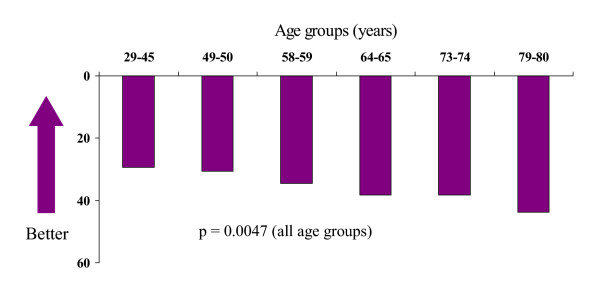
**SGRQ, Total score (mean values) in the six age groups. **p-values by test for trend.

The level of SF-36 PCS varied in the age groups from 44 ( < 45 years) to 36 ( > 79 years), with no statistical significance between the age groups. The level of SF-36 MCS was somewhat higher, 56 in the low age group and 51 in the high age group (not significant). The scores for SGRQ varied from 29 ( < 45 years) to 44 ( > 79 years) and they were statistically significant (p = 0.0047) (Figure 2). The scores for EQ-5D VAS varied as follows: 86 ( < 45 years) to 81 ( > 79 years). No statistical difference in EQ-5D VAS and EQ-5D index between the age groups could be seen.

The gender comparison showed only a statistically significant difference in SF-36 PCS, with scores of 44 for the men and 35 for the women (p = 0.0005).

The mean scores for SGRQ Total were 26, 36 and 31 in the non-smoker, ex-smoker and smoker groups, respectively (not significant).

No significant differences were seen in the other two instruments. Socio-economic group showed no difference for any instrument.

### HRQL in relation to FVC % predicted

The four stages of FVC % predicted ( > 95%, 95-81%, 80-66%, < 66%) had an impact on HRQL similar to the stages of FEV_1 _% predicted outlined from GOLD and BTS. SGRQ total score varied from 26 ( > 95%) to 43 ( < 66%) (p = 0.0002) (Table 5). Using the GOLD stages, the number of patients was unequally distributed and the SGRQ Total scores were 26 ( > 80%, n = 81), 40 (79-50%, n = 68) and 46 (49-30%, n = 10) (p < 0.0001). No patient had a value less than 30% predicted.

**Table 5:** Health-related quality of life scores, adjusted mean values (± SD) using FVC% predicted normal value

**Scale**	**FVC % predicted**				
	** > 95% n = 35**	**95-81% n = 33**	**80-66% n = 40**	** < 66% n = 34**	**p-value (all stages)**
SF-36 PCS	44(11)	45(11)	38(12)	35(10)	0.0008
SF-36 MCS	53(9)	52(10)	53(11)	49(14)	0.28
SGRQ Total	26(17)	29(17)	37(22)	43(20)	0.0002
EQ-5D VAS	71(19)	69(24)	63(25)	49(25)	0.0002
EQ-5D index	0.79(0.18)	0.80(0.19)	0.71(0.27)	0.62(0.26)	0.0017

### Correlations between the instruments

Table 6 shows the Pearson correlation coefficients between the different instruments and FEV_1 _% and FVC % predicted. All the questionnaires were correlated with each other. The correlation coefficients between SGRQ and SF-36 PCS/MCS were -0.62 and -0.42, respectively. The lowest correlation was seen between SF-36 MCS and SF-36 PCS (r = 0.22). The correlations between SGRQ and either FEV_1 _% predicted or FVC % predicted were similar (-0.34 and -0.37, respectively).

**Table 6:** Pearson's correlation coefficients (r)

	**SF-36 PCS**	**SF-36 MCS**	**SGRQ Total**	**EQ-5D VAS**	**EQ-5D index**	**FEV_1 _% predicted**	**FVC % predicted**
**SF-36 PCS**	1.0	--	--	--	--	--	--
**SF-36 MCS**	0.22	1.0	--	--	--	--	--
**SGRQ Total**	-0.62	-0.42	1.0	--	--	--	--
**EQ-5D VAS**	0.73	0.49	-0.63	1.0	--	--	--
**EQ-5D index**	0.64	0.58	-0.61	0.68	1.0	--	--
**FEV_1 _% predicted**	0.30	0.10	-0.34	0.38	0.26	1.0	--
**FVC % predicted**	0.32	0.08	-0.37	0.36	0.22	0.81	1.0

## Discussion

The present study confirms that disease severity (based on FEV_1_) and age influenced HRQL among subjects with COPD. HRQL was strongly related to impaired FEV_1 _in our study, which is in contrast to some previous studies [21]. The relationship between disease severity using FEV_1_% predicted and HRQL was made obvious by staging the disease according to the GOLD and BTS guidelines. Once COPD has been diagnosed, neither gender, smoking status nor socio-economic group predicted the level of HRQL.

The relationship between disease severity and HRQL across different chronic conditions, such as ischemic stroke, Parkinson's disease and coronary heart disease, has been examined [22]. It was concluded that in Parkinson's disease the relationship between disease severity and HRQL is linear, whereas in other diseases, such as chronic coronary heart disease, a non-linear relationship was observed. One of the most important implications of a non-linear relationship is that similar changes in disease severity may have a different effect on measured HRQL. Comparing other studies with the present results, some results highlight the fact that physical functioning is one of the most important predictors of HRQL in older subjects. The present results add the clinical value of multidimensional and complex measures of HRQL as previously described [23]. A moderate association between HRQL and COPD severity stage using FEV_1 _% predicted was seen in another study; however, a large variation in deterioration was observed within each stage of severity, indicating that both clinical and HRQL measures should be considered in the assessment of these patients [24]. In a study by Mahler et al, the decline in lung function over time may predict various components of general HRQL [25].

On the other hand, only a few studies have highlighted a relationship between disease severity and HRQL in COPD. A recent publication supports our findings by showing that GOLD stages of COPD severity differ significantly in SGRQ [26]. However, it was observed that the upper limit of stage IV marks a threshold for dramatic worsening of HRQL, whereas a change from stage 0 to II does not correspond to any meaningful difference in HRQL.

A moderate relationship between the disease stage of COPD and HRQL was found [27]. Our findings confirm these results as patients with COPD have significant decreases in HRQL, and the latter deteriorates in parallel with lung function impairment. An observational study was conducted to explore the relationship between various determinants of disease severity and HRQL [28]. According to its results, lung function and HRQL express several different aspects of disease severity in COPD.

As was found in a study of asthma [29], no gender difference was seen in our study. However, this is not always the case, as women tend to be more sensitive to changes in HRQL [30].

Smoking status did not affect the subjects' HRQL in the present study once COPD had been established. There are various results for the association between smoking status and HRQL. One study showed that COPD patients who continue smoking have a significantly lower HRQL than those who quit smoking [31]. On the other hand, current smoking has been associated with a better HRQL in the study by Wijnhoven et al. [28]. The explanation given was that subjects who do not quit smoking might be those with a less severe stage of disease. One limitation with the present results might be the low number of subjects in the very severe stage group, however, the ANCOVA analysis compensate for the skew distribution.

The correlations between lung function and HRQL have been shown to be weak in a number of studies [21]. In the present study the SGRQ Total score ranged from 23 in GOLD stage I to 56 in GOLD stage IV (according to BTS 23–47). The correlation between FEV_1 _% predicted and the HRQL measurement varied between -0.34 and 0.10, with the highest correlation (-0.34) between FEV_1 _% predicted and SGRQ Total score. One reason for the difference in correlation between lung function and HRQL may be the influence of psychosocial variables on the HRQL outcome. The subjects in our study seemed to score their HRQL better compared to other subject groups with similar lung function. One study supported the view that the association between lung function and HRQL can be predicted by perceived self-efficacy for functional activities [32]. That study suggested that both biomedical and psychosocial influences should be taken into account in order to provide optimum assessment and treatment. The correlations in this study were stronger than previous seen and another reason might be that disease severity was considered as a category rather than a continuous variable.

Using FVC % predicted did not add any additional information; however, it supported the view that the level of lung function measured by volume has a similar but lower association with HRQL compared with FEV_1 _in subjects with COPD.

## Conclusion

In conclusion, the results show that the level of health-related quality of life of COPD subjects deteriorates considerably with increasing severity of disease and that the deterioration is linearly related to a decrease in FEV_1 _% predicted normal values. A higher age also affected the COPD subjects' HRQL, while gender, smoking status and socio-economic group did not, once COPD had been established.

## Authors' contributions

Elisabeth Ståhl participated in the study design, evaluation of results and drafted the manuscript

Anne Lindberg, provided with subjects

Sven-Arne Jansson performed the interviews with the subjects

Eva Rönmark provided with subjects

Klas Svensson performed the statistical analysis

Fredrik Andersson participated in the study design

Claes-Göran Löfdahl gave support with interpretation of the results

Bo Lundbäck participated in the study design and responsible for the OLIN studies

All authors read and approved the final manuscript
